# Seeing emotions in the eyes – inverse priming effects induced by eyes expressing mental states

**DOI:** 10.3389/fpsyg.2014.01039

**Published:** 2014-09-17

**Authors:** Caroline Wagenbreth, Julia Rieger, Hans-Jochen Heinze, Tino Zaehle

**Affiliations:** Department of Neurology, Otto-von-Guericke University MagdeburgMagdeburg, Germany

**Keywords:** affective priming, lexical decision task, read the mind in the eyes, valence, face

## Abstract

**Objective:** Automatic emotional processing of faces and facial expressions gain more and more of relevance in terms of social communication. Among a variety of different primes, targets and tasks, whole face images and facial expressions have been used to affectively prime emotional responses. This study investigates whether emotional information provided solely in eye regions that display mental states can also trigger affective priming.

**Methods:** Sixteen subjects answered a lexical decision task (LDT) coupled with an affective priming paradigm. Emotion-associated eye regions were extracted from photographs of faces and acted as primes, whereas targets were either words or pseudo-words. Participants had to decide whether the targets were real German words or generated pseudo-words. Primes and targets belonged to the emotional categories “fear,” “disgust,” “happiness,” and “neutral.”

**Results:** A general valence effect for positive words was observed: responses in the LDT were faster for target words of the emotional category happiness when compared to other categories. Importantly, pictures of emotional eye regions preceding the target words affected their subsequent classification. While we show a classical priming effect for neutral target words – with shorter RT for congruent compared to incongruent prime-target pairs- , we observed an inverse priming effect for fearful and happy target words – with shorter RT for incongruent compared to congruent prime-target pairs. These inverse priming effects were driven exclusively by specific prime-target pairs.

**Conclusion:** Reduced facial emotional information is sufficient to induce automatic implicit emotional processing. The emotional-associated eye regions were processed with respect to their emotional valence and affected the performance on the LDT.

## INTRODUCTION

Due to the important role of facial features in social communication, effects arising from automatic affective processing of faces and facial expressions gain more and more of interest (Li et al., 2008; Suslow et al., 2010; Andrews et al., 2011; Rohr et al., 2012; Sassi et al., 2014). Faces are complex, concrete and socially significant stimuli that are linked to emotional reactions (Murphy and Zajonc, 1993). In addition, a human face holds a natural salience, i.e., it is an interesting stimulus that attracts attention more than other visual stimuli (Krebs et al., 2011). A faster detection of fearful or angry when compared to neutral faces (Ishai et al., 2004) as well as an attentional bias to threatening facial expressions could be observed (Susa et al., 2012). Moreover, a facilitation of a visual search task to identify fear-related pictures among fear-irrelevant ones was shown (Öhman et al., 2001) as well as a slower attention disengagement from angry faces compared to neutral or happy ones (Fox et al., 2002). In agreement with the evolutionary point of view this suggests a faster processing of threatening or possibly life-endangering stimuli. Thus, decoding and interpreting emotions displayed in facial expressions, especially those with negative valence, play a fundamental role in human interactions.

Identifying emotional states of other persons also constitutes the basis of the social construct of theory of mind (ToM). ToM refers to the process of emotion recognition and allows individuals to imagine and attribute the mental states of others (Pinkham et al., 2003). The “Reading the mind in the eyes task” (RMET) which was invented by Baron-Cohen et al. (1997) is an advanced test of ToM and consists of photographs showing the facial region around the eyes. Four different emotional states are offered and the participant is asked to decide which of these emotional states describes best what the person on the photograph is thinking or feeling. Thus, the RMET is a social cognition measure to evaluate the participant′s ability to recognize and identify mental states and their intensity. It refers to explicit emotional processing which requires conscious action under deliberate control. In contrast, implicit processing of emotions is an automatic process that does not allow for the activation of expectancies or response strategies and is thus independent of cognitive resources. Implicit – or automatic – activation of emotional evaluations is often measured in affective priming paradigms (Fazio, 2001; Klauer and Musch, 2003). Here, the automatically triggered connection between two stimuli (prime and target) which are determined through their emotional valence is essential. Faster responses and fewer errors were observed when two consecutively presented stimuli are identical (congruent) with respect to their emotional valence (e.g., positive–positive) than when they differ from each other (Andrews et al., 2011). This congruence effect has been shown to be a robust and replicable phenomenon in a variety of studies using different primes, targets and specific instructions and tasks (Hermans et al., 1994, 1998, 2003; De Houwer et al., 1998; Giner-Sorolla et al., 1999; Banse, 2001). The effect of congruence between prime and target allows for the systematic evaluation of the automatic emotional processing that is triggered by the prime.

In affective priming paradigms, the most commonly used task for the participants is to evaluate a target concerning its valence, as applied in a lexical decision task (LDT). The LDT is a common instrument to measure implicit emotional processing since it focuses on a non-emotional decision which is not distorted by emotional features of the stimuli. In a LDT, participants have to judge the lexical status of a presented letter string on whether it is a correct real word or a pseudo- or nonword. To ensure an “affective” influence on the LDT, words with different emotional valences are used in this task. Hence, valence is an important manipulating variable on the performance in LDTs. Different studies showed shorter reaction times (RT) and fewer errors of participants for emotional when compared to neutral words (Challis and Krane, 1988; Williamson et al., 1991; Windmann et al., 2002; Nakic et al., 2006; Schacht and Sommer, 2009). Positive valence is known to facilitate lexical processing in the LDT (Kuchinke et al., 2005, 2007; Schacht and Sommer, 2009; Scott et al., 2009) while negative valence itself seems to slow RT (Briesemeister et al., 2011a). This effect on RT for negatively valenced words is observed only at high levels of emotional arousal (Nakic et al., 2006; Larsen et al., 2008; Hofmann et al., 2009), which suggests a simplified and preferred processing of for instance fearful words that are expected to have a high emotional arousal.

In recent studies that applied affective priming paradigms, whole face images and facial expressions have been used as primes since facial features are supposed to be recognized ubiquitously as indicators of different affective conditions (Li et al., 2008; Suslow et al., 2010; Andrews et al., 2011; Rohr et al., 2012; Sassi et al., 2014). However, it is not clear whether these findings are also true for reduced emotional information that is featured not in the whole face, but solely in the eye regions of a face expressing mental states. Therefore, in the present study we systematically investigated the ability of emotional eyes expressions to induce implicit emotional processing by using an affective priming paradigm. We assume that even reduced emotional information in human eyes is sufficient to influence automatic emotional responses. Here, eyes expressing mental states acted as primes to influence the subsequent answer on a target word in a LDT.

## MATERIALS AND METHODS

### SUBJECTS

Sixteen healthy participants (female = 8) in the age of 22–28 years (mean age = 24.12 ± 1.54 years) volunteered in this study. All participants were German native speakers, right-handed, reported normal, or corrected-to-normal vision and were free of any neurological or psychiatric impairment. The experiment was approved by the local ethics committee (University of Magdeburg, Germany).

### MATERIAL

For the LDT a set of 192 written stimuli (96 German words and 96 pseudo-words) were used. Neutral words were extracted from the “Berlin Affective Word List Reloaded” (BAWL-R; Võ et al., 2009) when they showed a valence rating of 0. Emotional words were taken from the “discrete emotion norms for nouns: Berlin affective word list” (DENN-BAWL; Briesemeister et al., 2011b). Here, only those words were chosen that achieved an emotional intensity score of at least 3 on a Likert-scale according to Briesemeister et al. (2011b). All 96 words were grouped in four emotional categories: 24 fear-related [e.g., “PANIK”; (“PANIC”)], 24 disgust-related [e.g., “PARASIT”; (“PARASITE”)], 24 happiness-related [e.g., “LIEBE”; (“LOVE”)] and 24 neutral words [e.g., “WOCHE”; (“WEEK”)]. Pseudo-words were also generated out of the words listed in the BAWL-R. Here, neutral words with a length of four to eight letters were manipulated by interchanging single vowels or consonants within this word. By doing so, new pronounceable but meaningless letter strings were created (e.g., “POLITIK” – “PILITOK”).

Facial region around the eyes extracted from the stimulus set “60 Faces Test” developed by Ekman and Friesen (1976) were used as emotional primes. This set consists of the six basic human emotional facial expressions (happiness, sadness, disgust, fear, surprise, and anger as well as a neutral condition). For the present study, we chose four different persons out of this set (two female, two male) and selected their relevant facial expressions on a picture (happiness, fear, disgust, neutral). The whole-face images were then adjusted and cut so that only the eye regions were visible. After this editing, the resulting 16 prime pictures were presented in a size of 8.6 cm × 3 cm (3.4 visual angle).

Primes (eye region pictures) and target stimuli (words or pseudo-words) were combined in a pseudo-randomized manner: half of the prime-target pairs of the real German words were constructed to be congruent. The sequence of combination is displayed in **Table 1**.

**Table 1:** Division of the prime-target word-pairings in the lexical decision task (LDT) according to congruence.

Congruence	Combinations		Number per pair	Total number
	Prime	Target word		
Congruent	Fear	Fear	12	48
	Disgust	Disgust	12	
	Happiness	Happiness	12	
	Neutral	Neutral	12	
Incongruent	Fear	Disgust	4	48
	Fear	Happiness	4	
	Fear	Neutral	4	
	Disgust	Fear	4	
	Disgust	Happiness	4	
	Disgust	Neutral	4	
	Happiness	Fear	4	
	Happiness	Disgust	4	
	Happiness	Neutral	4	
	Neutral	Fear	4	
	Neutral	Disgust	4	
	Neutral	Happiness	4	

### TASK

The experiment was generated and carried out with presentation (Neurobehavioral Systems, Inc.). Participants were seated in front of a computer and were requested to look at a fixation cross in the middle of the screen. The task consisted of 192 trials and included a break after the half of the trials (96); the participants could decide how long this break would last. Depending on the understanding of instruction and the length of the break, the duration of the whole task was about 15 min.

**FIGURE 1:**
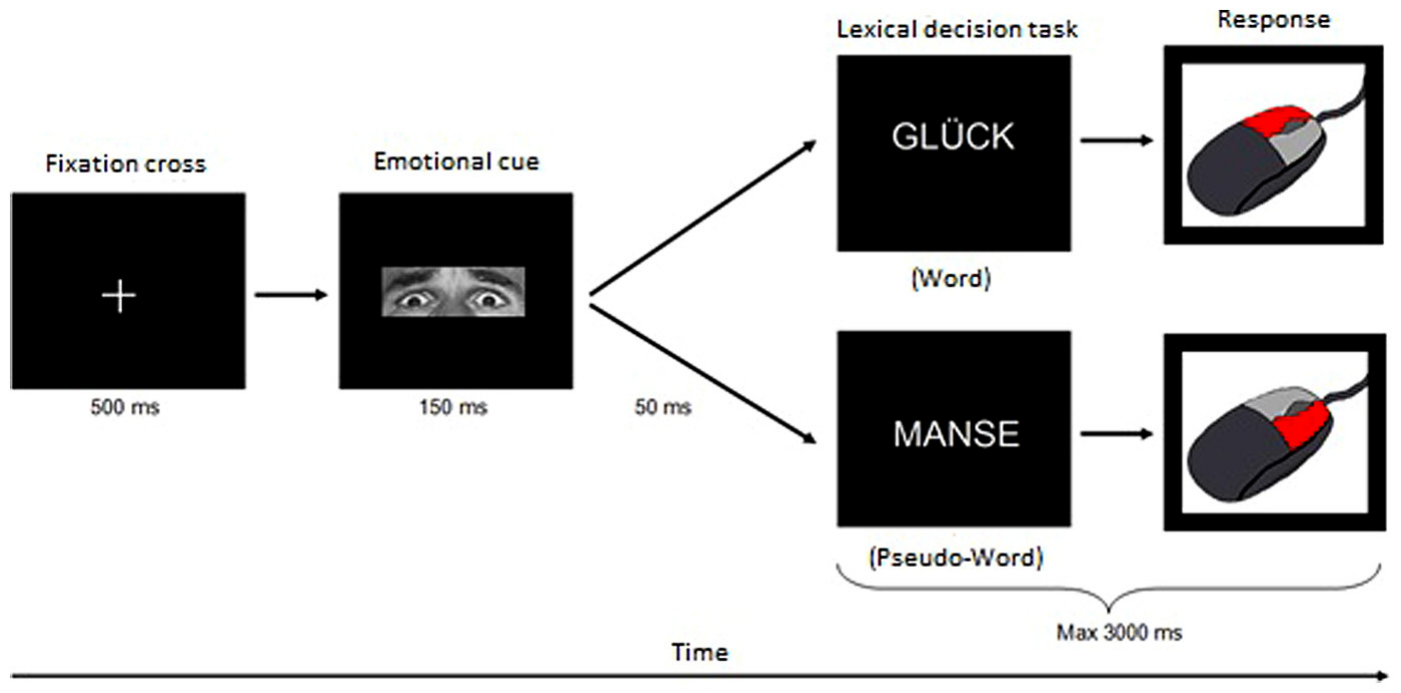
**Experimental design of the implicit emotional processing with LDT.** Each trial started with a fixation cross that was presented for 50 ms. Subsequently an emotional prime (the adjusted eye region of the Ekman-faces) was displayed for 150 ms followed by a break of 50 ms. Finally, the target stimulus was presented. The participants were supposed to decide as fast as possible whether this target was a real German word or a pseudo-word and to press the corresponding button on the mouse within 3000 ms. After this time interval the next trial started.

Each trial started with a fixation cross that was shown for 500 ms before an emotional prime was displayed for 150 ms. After a short pause (inter-stimulus interval of 50 ms) the target stimulus was presented for a maximum of 3000 ms or until a response was made. During this time interval, the participants had to decide whether this target was a real word or a pseudo-word by pressing the corresponding button on the mouse. Afterward, the next trial started (see **Figure 1**). The presentation order of items (primes and targets) as well as the assignment of mouse buttons to respond was counterbalanced across subjects.

## ANALYSIS

Mean RT and error rates for each participant were assessed and entered into a 4 × 2 repeated-measures ANOVA with the factors *valence* of the target word (fear, disgust, happiness, neutral) and emotional *congruence* of the prime-word pairing (congruent, incongruent). Subsequently individual prime-target pairings were evaluated separately. Prior to analysis RT normal distribution was confirmed using the Kolmogorov–Smirnov Test of Normality (*p* = 0.89). All results are displayed in mean ± SD (M ± SD).

## RESULTS

**FIGURE 2:**
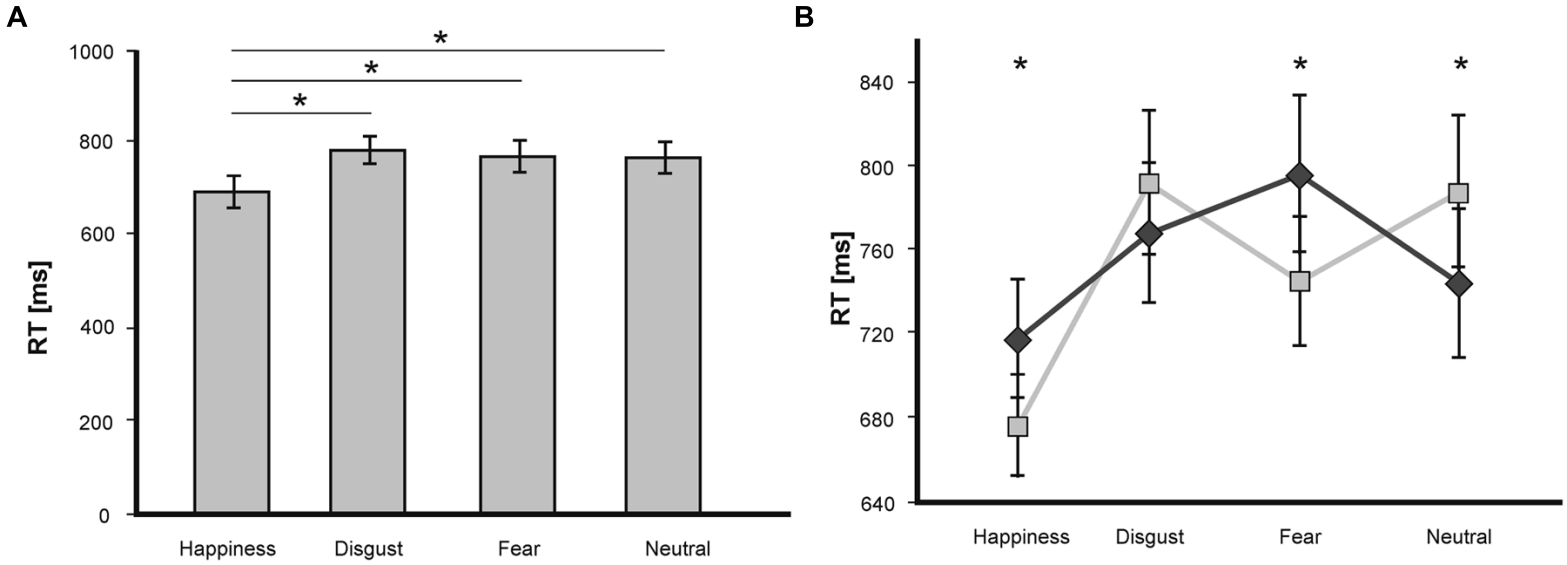
**(A)** Mean RT (in ms) in the LDT for target words of the emotional categories fear, disgust, happiness and neutral. Statistically significant differences are labeled with (*). Error bars display SEs. **(B)** Mean RT (in ms) in the LDT as a function of valence and congruence. Statistically significant differences are labeled with (*). Dark gray line depicts congruent trials, light gray line depicts incongruent trials.

A repeated-measures ANOVA on RT showed a significant main effect of the factor *valence* [*F*(3,45) = 14.84; *p* < 0.001], no main effect of the factor *congruence* [*F*(1,15) = 0.27; *p* = 0.61], and a significant *valence* ×*congruence* interaction [*F*(3,45) = 9.04; *p* < 0.001]. The main effect of *valence* was driven by significantly shorter RT for happiness-related target words compared to all other emotional categories (all *p* < 0.001; see **Figure 2A**).

The *valence* x *congruence* interaction was further investigated by comparing incongruent and congruent trials separately for each emotional target word category. Pairwise *t*-tests revealed shorter RT for incongruent prime-target pairs compared to congruent pairs for the word categories fear [*T*(15) = 2.35; *p* < 0.05] and happiness [*T*(15) = 2.67; *p* < 0.05], and shorter RT for congruent pairs compared to incongruent pairs [*T*(15) = -2.66; *p* < 0.05] for neutral targets (see **Figure 2B**). No effects were visible for the emotion category disgust [*T*(15) = -1.08; *p* = 0.3]. Thus, whereas there was a classical priming effect for neutral target words with a processing advantage for congruent pairs, we revealed an inverse priming effect for fearful and happy target words with an advantage for the processing of incongruent primed targets.

**FIGURE 3:**
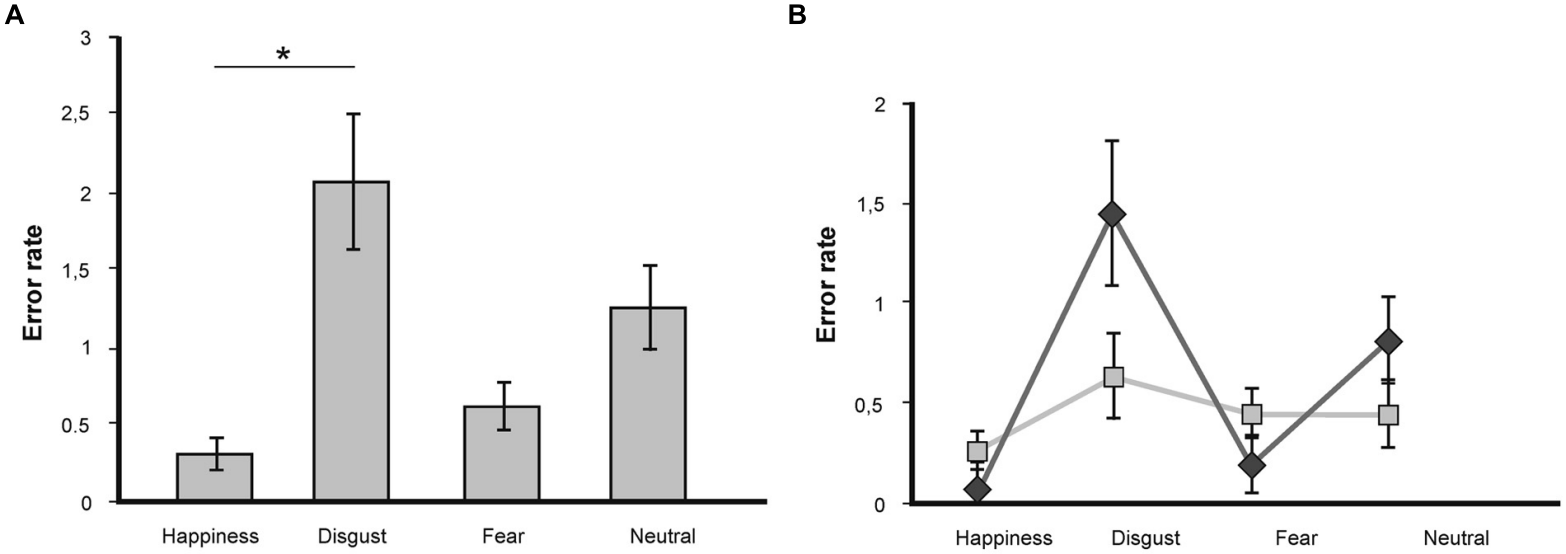
**(A)** Mean number of errors in the LDT for target words of the emotional categories fear, disgust, happiness, and neutral. Statistically significant differences are labeled with (*). Error bars display SEs. **(B)** Mean number of errors in the LDT as a function of valence and congruence. Statistically significant differences are labeled with (*). Dark gray line depicts congruent trials, light gray line depicts incongruent trials.

A repeated-measures ANOVA on error rate showed a non-significant statistical trend for the factor *valence* [*F*(3,45) = 2.44; *p* = 0.07], no effect of the factor congruence [*F*(1,15) = 1.36; *p* = 0.26], and no significant *valence* x *congruence* interaction [*F*(3,45) = 0.67; *p* = 0.57]. The trend for the main effect of *valence* was driven by significantly more errors for disgust-related words compared to happiness-related words (*p* = 0.05; see **Figure 3A**).

Further analysis showed no differences in error rate for incongruent versus congruent conditions for the emotional word categories (happiness: [*T*(15) = 0.44; *p* = 0.67]; fear: [*T*(15) = 0.0; *p* = 1.0]; disgust: [*T*(15) = 0.22; *p* = 0.83]; neutral [*T*(15) = 1.86; *p* = 0.08]; see **Figure 3B**).

**FIGURE 4:**
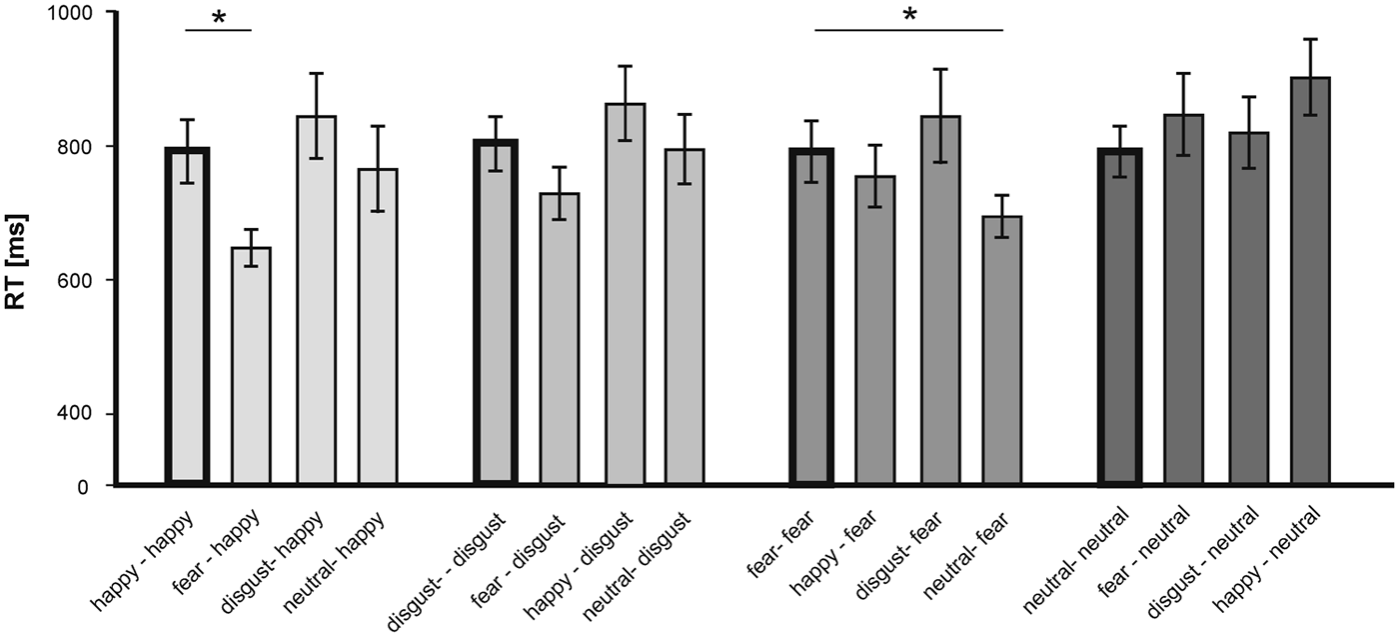
**Mean RT (in ms) in the LDT for single emotional prime-target pairings according to the valence of the target.** Statistically significant differences are labeled with (*). Error bars display SEs.

To further assess the observed priming effects, we analyzed responses to individual primes-targets pairs separately (see **Figure 4**).

For happy target words, analysis revealed that compared to the congruent (happy–happy) pairs only the incongruent fear-happy pairs was answered significantly faster [*T*(15) = - 5.73; *p* < 0.001]. For fearful target words, analysis showed that the neutral-fear pairs were answered significantly faster that the congruent (fear–fear) pairs [*T*(15) = -4.62; *p* < 0.001; see **Figure 4**]. No further congruent–incongruent comparison revealed statistical significance.

In sum, the valence of the target words had a significant effect on the RT in the LDT. Happiness-related target words were answered faster than words of all other emotional categories. Importantly, the emotional content of the facial expressions (primes) modulated the LDT responses. While we could show a classical priming effect for neutral target words – with shorter RT for congruent compared to incongruent prime-target pairs- , we observed an inverse priming effect for fearful and happy target words – with shorter RT for incongruent compared to congruent prime-target pairs. Furthermore, this inverse priming effect for happy targets was driven exclusively by the incongruent combination with fearful primes, whereas for fearful targets it was driven by neutral primes only.

## DISCUSSION

### EFFECT OF VALENCE

Independent from the prime-target pairing, we found a general effect of valence in the LDT with shorter RT for emotional target words when compared to neutral words. Thus, an advantage in the processing of positive words (category happiness) in form of shorter RT when compared to fearful, disgust-related or neutral words was observed. However, the other categories (fear, disgust) did not differ significantly from neutral words. These findings are in good agreement with previous studies that postulate an advantageous processing of positive words (Kuchinke et al., 2005; Hofmann et al., 2009; Briesemeister et al., 2011a; Kissler and Koessler, 2011). Slowing in RT for the processing of negative words has been described recently (Wentura et al., 2000; Castner et al., 2007). In our study this increase of RT for negative words was significant when compared to positive, but not when compared to neutral words. These findings can be attributed to the “automatic vigilance” hypothesis implying that people tend to focus their attention preferentially on negative stimuli and can also rather difficult dissolve it from them (Wentura et al., 2000; Öhman and Mineka, 2001). This is in line with findings by Estes and Verges (2008) who postulated a slower disentanglement of attention from negative stimuli and with an approach by Larsen et al. (2008) who suggest a RT slowing for low arousing negative words.

### EFFECT OF CONGRUENCE

We observed an interaction effect between valence and congruence which demonstrates that the influence of emotional congruency depends on the valence of the target word. A general effect of congruence between prime and target, displayed in shorter RT and fewer errors in congruent when compared to incongruent trials, is often reported in affective priming paradigms (Banse, 2001; De Houwer et al., 2009). Interestingly, the present results confirm this effect only in part. The advantage of affectively congruent primed trials was found for neutral words only and selectively for RT and not for the error rate. Contrary to the expectations, we found an *inverse priming effect i*n terms of an advantageous processing of incongruent trials: for the emotional categories fear and happiness RT were shorter for incongruent than for congruent trials.

Different explanations have been suggested for the inverse priming effect (Glaser and Banaji, 1999; Banse, 2001; Verleger et al., 2004; Mattler, 2007). Glaser and Banaji (1999) underline the modulating character of the extremeness of primes that would provoke an automatic and unconscious correction of information processing. Following their argumentation, primes of extreme valence have the potential to distort the answer on a target. Thus, the emotional processing of “extremely valenced” primes (e.g., fearful or happy) would be increased and a proper lexical decision would require more time. In contrast, neutral primes have lower salience and no correction of distorting influences is necessary, which is reflected in shorter RT. Critically, this approach highlights the importance of the emotional information content of the primes. However, the role of the valence of targets for emotional processing is not affected and thus this explanation does not sufficiently account for the present findings.

Another approach is given by Maier et al. (2003). They suggested the existence of activation dependent inhibitory processes in memory. According to the authors, a strengthening of some examples of a category (i.e., either positive or negative) is connected with an automatic inhibition of related, but interfering examples of the same category. For instance, as soon as the representation of a positive stimulus as “positive” is enhanced, automatic activation processes start inhibiting stimuli of the same category (“positive”). Effects of congruency would lose ground in favor of automatic inhibitory processes and the accessibility for an incongruent target word would thus increase. This could explain RT advantages of incongruent pairs of the category happiness and fear, but not why RT are faster for incongruent neutral word trials.

Based on different explanatory attempts one might suggest a concluding approach that integrates different theories. The basic assumption here is that the valence of the primes and targets is processed unconsciously and that correction and inhibition processes are involved in affective priming. In this hypothesis, the presentation of an emotional prime activates the appropriate concept node in the semantic network (De Houwer and Hermans, 1994). According to Maier et al. (2003) the corresponding representation in the greater valence system (e.g., the concept “negative”) is enhanced through automatic activation dispersion. If a congruent target stimulus follows, a stronger activation of the belonging memory network is run due to the additive effect of the valence information of both prime and target (Dagenbach and Carr, 1994) which results in longer RT for congruent trials. For incongruent trials, both valence representations are activated in memory, but each system apart has only a low activation level. Thus, the automatic correction system for the distortive influence of emotional information (Glaser and Banaji, 1999; Klapp, 2005) can be inhibited in favor of an adequate response. Since the activation levels of both representations are low, the activation of both valence systems is faster which results in shorter RT for incongruent trials. Thus, the strength of the activation levels determines the strength of the activation of the automatic correction system which in turn is expressed in RT. For neutral words that do not carry emotional information, no activation of greater valence systems is necessary as well as no corrective efforts. Thus, RT for neutral congruent prime-target pairs (which have no influence of relevant emotional information) are shorter than RT of neutral incongruent pairs.

Beyond the general inverse priming effects for happiness and fear, the present data show that the processing advantage for incongruently primed happy targets was mainly driven by the fearful primes. The combination of fear (prime) and happiness (target) was answered faster than all other conditions. This implies a special role of fear as characteristic for a prime. In general, fear expressions have been shown to reveal stronger subliminal priming effects than other negative emotional expressions as for instance disgust (Murphy and Zajonc, 1993; Lee et al., 2011). Fear is thus suggested to activate subliminal processing as an alarm for possible threat. In our study, the pairing fear–happy was answered fastest. It might be suggested that the fear primes acted as threat signals and increased attentional vigilance. Thus, fearful faces are processed more quickly during encoding and are more available for subsequent processing (Suslow et al., 2006). However, since happy stimuli convey quite clear and undoubted safety signals, the emersion of a happy word might resolve the alertness and elicit faster RT.

But advantage in fear processing does not only apply to primes but also to targets. Interestingly, negative stimuli (such as fear-associated) are reported to elicit longer RT than positive ones (Castner et al., 2007). And in general, fearful expressions are detected more quickly than neutral or happy expressions (Yang et al., 2007; Wang et al., 2014). In our study, we detected a fast processing of fear target words that were preceded by neutral primes, which determined the general inverse priming effect for fearful targets. The neutral facial expression corresponds to the normal, everyday facial expression that one is most accustomed to when looking in faces of other persons. No emotional processing is necessary when assessing neutral expressions. We assume that the discrepancy between this neutral expression and a sudden appearance of a threatening fear target word leads to fast responses, especially when compared to other emotional facial expressions that always imply emotional anticipation.

Affective information is not distributed uniformly in the face. There is agreement that the recognition of fearful and angry faces depends more on information in the eye region, while disgust is conveyed mainly by the mouth (Calder et al., 2000; Leppänen and Hietanen, 2007; Calvo and Marrero, 2009). In contrast to the emotions fear or happiness, the emotional category disgust captures an exceptional position. When analyzing the influence of the targets’ valences on performance, most errors were detected for disgust-related target words. At the same time, no differences between congruent and incongruent conditions for disgust target words could be detected. Thus, for target words of this category no priming effect was present. Recent findings support the special role of disgust in comparison to other emotions (Sprengelmeyer et al., 1996; Phillips et al., 1997; Wang et al., 2003; Montagne et al., 2006; Hayes et al., 2007; Lee et al., 2011; Baggio et al., 2012; Borg et al., 2012; Buxton et al., 2013) indicating disgust as a negative but nonetheless non-threatening emotion in social contexts. Moreover, disgust is a highly-evaluative emotion that is predominantly affected through the culture a society lives with. Thus, there are considerable differences in what people sense or perceive as “disgusting.” The choice of disgust-associated stimuli therefore constitutes a challenging task for affective priming paradigms to prevent ambiguity and future studies should consider this point carefully.

### LIMITATIONS

Despite the strength of this study to demonstrate that eye regions of a face displaying mental states elicit affective priming, some confining points have to be taken into account. We present data from a rather small and very homogeneous sample concerning age and educational background. Future studies should consider more variability to report different characteristics of affective priming during implicit emotional processing. Moreover, the words used in this investigation were not selected with respect to an equally distributed word frequency. We cannot exclude possible influences of this variable on the results. However, recent studies could demonstrate RT advantage of positive words independent from the word frequency (Kuchinke et al., 2007) which underlines our findings.

## CONCLUSION

To our knowledge, this is the first study demonstrating that even incomplete facial information induce implicit emotional responses and – in consequence – influence subsequent explicit decisions. These emotion-associated eye regions were processed with regard to their emotional valence and affected the performance on the following LDT. The primes used here clearly demonstrated their effectiveness in affective priming.

## Conflict of Interest Statement

The authors declare that the research was conducted in the absence of any commercial or financial relationships that could be construed as a potential conflict of interest.
